# FTO Positively Regulates Odontoblastic Differentiation via SMOC2 in Human Stem Cells from the Apical Papilla under Inflammatory Microenvironment

**DOI:** 10.3390/ijms25074045

**Published:** 2024-04-05

**Authors:** Qi Huang, Yumei Sun, Wushuang Huang, Fuping Zhang, Hongwen He, Yifan He, Fang Huang

**Affiliations:** 1 Hospital of Stomatology, Sun Yat-sen University, Guangzhou 510055, China; huangq257@mail2.sysu.edu.cn (Q.H.); 13667453885@163.com (Y.S.); huangwsh26@mail.sysu.edu.cn (W.H.); zhangfp6@mail.sysu.edu.cn (F.Z.); hehw@mail.sysu.edu.cn (H.H.); 2 Guangdong Provincial Key Laboratory of Stomatology, Guangzhou 510055, China; 3 Guanghua School of Stomatology, Sun Yat-sen University, Guangzhou 510055, China

**Keywords:** fat mass and obesity-associated protein, human stem cells from the apical papilla, odontoblastic differentiation, secreted modular calcium-binding protein 2, inflammation

## Abstract

Odontoblastic differentiation of human stem cells from the apical papilla (hSCAPs) is crucial for continued root development and dentin formation in immature teeth with apical periodontitis (AP). Fat mass and obesity-associated protein (FTO) has been reported to regulate bone regeneration and osteogenic differentiation profoundly. However, the effect of FTO on hSCAPs remains unknown. This study aimed to identify the potential function of FTO in hSCAPs’ odontoblastic differentiation under normal and inflammatory conditions and to investigate its underlying mechanism preliminarily. Histological staining and micro-computed tomography were used to evaluate root development and FTO expression in SD rats with induced AP. The odontoblastic differentiation ability of hSCAPs was assessed via alkaline phosphatase and alizarin red S staining, qRT-PCR, and Western blotting. Gain- and loss-of-function assays and online bioinformatics tools were conducted to explore the function of FTO and its potential mechanism in modulating hSCAPs differentiation. Significantly downregulated FTO expression and root developmental defects were observed in rats with AP. FTO expression notably increased during in vitro odontoblastic differentiation of hSCAPs, while lipopolysaccharide (LPS) inhibited FTO expression and odontoblastic differentiation. Knockdown of FTO impaired odontoblastic differentiation, whereas FTO overexpression alleviated the inhibitory effects of LPS on differentiation. Furthermore, FTO promoted the expression of secreted modular calcium-binding protein 2 (SMOC2), and the knockdown of SMOC2 in hSCAPs partially attenuated the promotion of odontoblastic differentiation mediated by FTO overexpression under LPS-induced inflammation. This study revealed that FTO positively regulates the odontoblastic differentiation ability of hSCAPs by promoting SMOC2 expression. Furthermore, LPS-induced inflammation compromises the odontoblastic differentiation of hSCAPs by downregulating FTO, highlighting the promising role of FTO in regulating hSCAPs differentiation under the inflammatory microenvironment.

## 1. Introduction

Apical periodontitis (AP) in immature permanent teeth is a prevalent oral disease among pediatric patients, which inevitably disrupts root development, impedes apical foramina closure, and threatens the long-term survival of teeth. While apexification and regenerative endodontics are widely applied in clinical practice, histological evidence supporting genuine dentin–pulp regeneration remains inconclusive. The regenerated tissue resembles ectopic periodontal ligaments and cementum-like or bone-like structures [1,2,3,4]. Such tissue discrepancy emphasizes the gaps in understanding the mechanisms modulating stem cell responses in infected young teeth.

Different stem cells localized in the residual vital pulp, apical papilla, and periodontal tissue into odontoblast-like cells are central to root dentin regeneration and repair. Among them, human stem cells from the apical papilla (hSCAPs) have emerged as the most promising stem cell source for regenerative endodontics since they are in particular proximity to developing teeth apices and exhibit superior mineralization potential and proliferation rates compared with dental pulp stem cells (DPSCs) and periodontal ligament stem cells (PDLSCs) [5,6]. Inflammation or injuries often compromise stem cell functionality, impeding effective tissue regeneration and repair. While prior investigations have emphasized that the compromised odontoblastic differentiation ability of hSCAPs is closely associated with root development arrest in AP, the underlying mechanisms remain unclear [7,8,9,10]. Exploring the specific molecular regulatory pathway governing hSCAPs’ odontoblastic differentiation under physiological and pathological conditions holds significance for achieving sustained root development and enhancing dentinal tissues in immature teeth with AP.

N6-methyladenosine (m^6^A) is the most prevalent post-transcriptional modification of messenger RNA (mRNA) crucial for tooth development and odontoblastic differentiation of dental stem cells [11,12,13]. Fat mass and obesity-associated protein (FTO), identified as the first m^6^A effector, belongs to the AlkB-related superfamily, facilitating genome-wide m^6^A demethylation in an Fe (II)/α-ketoglutarate (α-KG)-dependent manner [14]. Evidence has emphasized that FTO is essential for craniofacial development and bone formation [15,16,17,18,19]. Recent findings discovered that FTO depletion results in dentine formation defects and impairs odontoblast differentiation of DPSCs [20]. Moreover, FTO promoted osteogenic and cementoblast differentiation through the HIF-1α, Runx2, p-AMPK, and PPARG pathways [21,22,23,24]. FTO deficiency accelerated the senescence of human mesenchymal progenitor cells and diminished their self-renewal potential [25]. The dynamic influence of FTO on the proliferation and differentiation of adult neural stem cells has been observed [26]. However, the specific role of FTO in hSCAPs differentiation has seldom been reported.

Secreted modular calcium-binding protein 2 (SMOC2), an extracellular glycoprotein belonging to the secreted protein acidic and rich in cysteine (SPARC) family, has been identified as a pathogenic gene for radicular dentin dysplasia [27]. Extensive in vivo studies have confirmed the close association of SMOC2 with root dentin formation. Individuals with SMOC2 mutations manifested severe microdontia, oligodontia, and dysplastic root formation [28,29,30,31], while SMOC2^−/−^ mutant mice exhibited spontaneous alveolar bone degradation and root resorption [32]. However, the molecular signaling mechanisms regulating SMOC2 during dentinogenesis have rarely been studied. SMOC2 has been reported to promote osteoblast differentiation and bone formation through Runx2 [33]. Additionally, a negative correlation between SMOC2 expression and m^6^A modification in fibroblast-like synoviocytes has been observed [34], suggesting the potential function of RNA modification in regulating SMOC2 expression. This study noticed a significant interaction propensity between FTO and SMOC2 utilizing online bioinformatics platforms. Subsequently, we explored whether SMOC2 functions as a downstream signaling molecule of FTO during hSCAPs differentiation.

This research aimed to investigate the involvement of FTO in immature teeth with AP and identified its specific role in hSCAPs’ odontoblastic differentiation. We analyzed root development and FTO expression alterations using an in vivo model. Subsequently, we assessed the impact of lipopolysaccharide (LPS) on odontoblastic differentiation and FTO expression in hSCAPs, elucidating FTO’s potential role through gain- and loss-of-function approaches. Furthermore, we preliminarily explored whether FTO modulates hSCAPs differentiation through SMOC2.

## 2. Results

### 2.1. FTO Significantly Downregulated in Immature Teeth with AP

**Figure 1:**
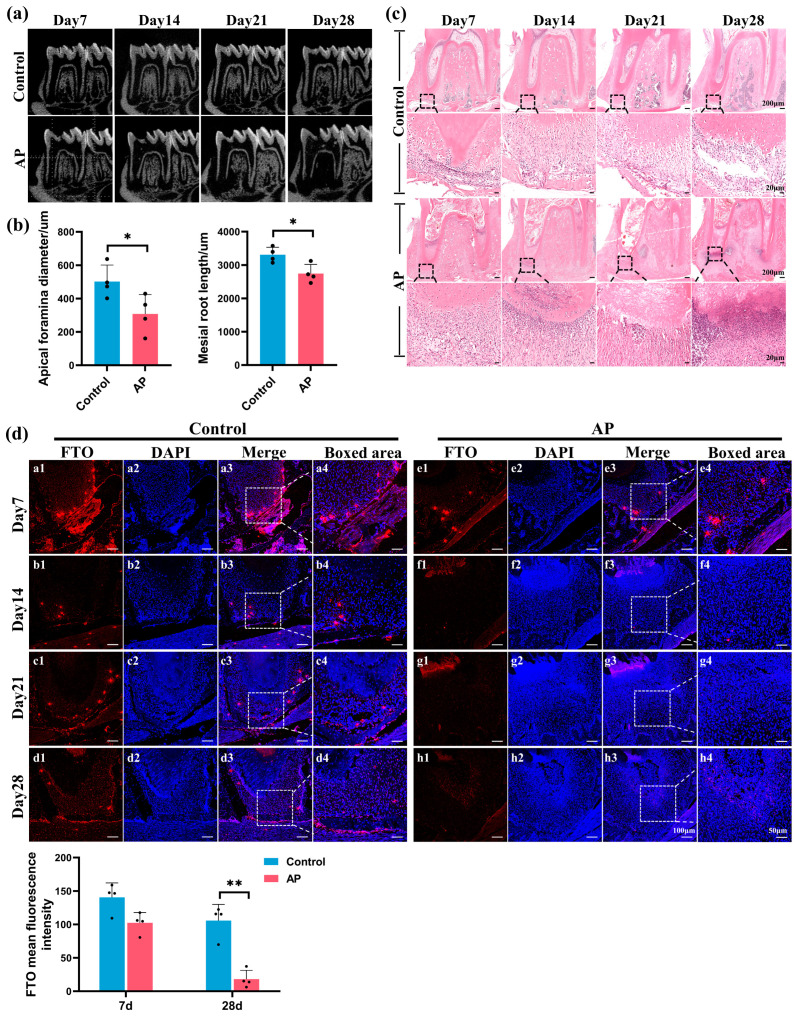
FTO significantly downregulated in the AP group’s apical region. (**a**) Micro-CT-validated AP models were successfully established. (**b**) Radiographic images on day 28 revealed the AP group’s expanded apical foramen and reduced mesial root length. (**c**) HE staining highlighted inflammatory cell infiltration and morphological alterations in the AP group’s apical region; images were magnified 8 times from areas enclosed by the black broken lines, respectively. (**d**) Immunostaining (10× magnification) and quantification demonstrated decreased anti-FTO staining levels in the AP group; images were magnified 2 times from areas enclosed by the white broken lines, respectively; the anti-FTO primary antibody was combined by DyLight 594-conjugated secondary antibody and the nucleus was stained by DAPI. FTO, fat mass and obesity-associated protein; AP, apical periodontitis; micro-CT, micro-computed tomography; HE, hematoxylin–eosin. * *p* < 0.05, ** *p* < 0.01. Error bars: mean ± SD.

The in vivo AP models were successfully established and validated using micro-computed tomography (micro-CT) and histological analyses of the mandibles in experimental rats. Radiographic analyses from days 7 to 28 revealed gradual enlargement of the lesion area adjacent to the mesial root of the mandibular first molar (Figure 1a). By analyzing radiographic data on day 28, the AP group displayed expanded apical foramen and reduced mesial root length compared to the control (Figure 1b). Hematoxylin–eosin (HE) staining highlighted the AP group’s marked inflammatory cell infiltration and tissue damage (Figure 1c). Immunofluorescence (IF) further demonstrated significant downregulation of the anti-FTO staining levels in the AP group’s apical area relative to the control (Figure 1d), suggesting its involvement in AP progression and potential influence on root development.

### 2.2. Characterization of hSCAPs and Establishment of In Vitro Model

**Figure 2:**
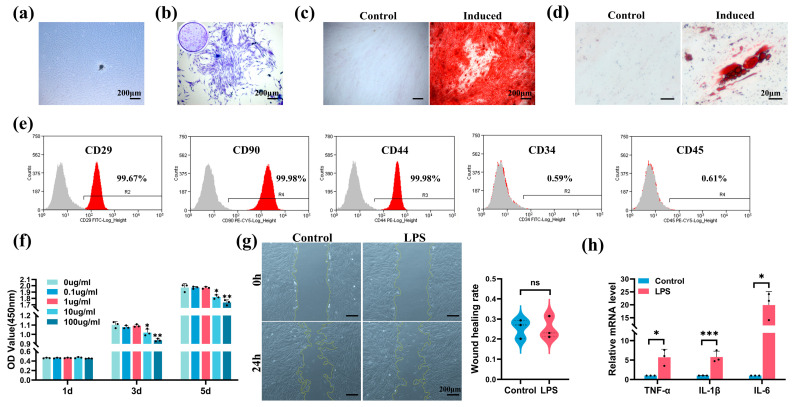
Characterization of hSCAPs and optimal LPS stimulation concentration determination. (**a**) Primary hSCAPs morphology on day 12. (**b**) Microscopic and macroscopic (blue circle) observations of crystal violet staining to demonstrate the colony-forming ability. (**c**) ARS staining showed mineralization nodules. (**d**) Oil red O staining indicated lipid droplets. (**e**) Flow cytometry showed hSCAPs positive for CD29, CD90, and CD44, and negative for CD34 and CD45; gray: number of cells labeled with isotype control antibodies that appear in the indicated fluorescence intensity range, red: number of cells labeled with antibodies of indicated makers that appear in the indicated fluorescence intensity range (**f**) CCK-8 depicted reduced hSCAPs viability with 10 and 100 µg/mL LPS for 3–5 days. (**g**) The wound healing assay revealed no significant difference in migration rates between the control (growth medium, GM) and LPS group (GM with 1 µg/mL LPS). (**h**) qRT-PCR confirmed significantly upregulated TNF-α, IL-1β, and IL-6 mRNA levels in the LPS group (GM with 1 µg/mL LPS) compared to the control (GM). hSCAPs, human stem cells from the apical papilla; LPS, lipopolysaccharide; ARS, alizarin red S; CCK-8, Cell Counting Kit-8; qRT-PCR, Quantitative Real-Time Polymerase Chain Reaction; TNF-α, tumor necrosis factor-alpha; IL-1β, interleukin-1beta; IL-6, interleukin-6. ns: *p* > 0.05, * *p* < 0.05, ** *p* < 0.01, *** *p* < 0.001. Error bars: mean ± SD.

The hSCAPs exhibited a typical spindle-shaped appearance (Figure 2a). Crystal violet staining underscored the colony-forming ability of hSCAPs (Figure 2b). After 21 days of odontoblastic and adipogenic inductions, noticeable mineralized nodules (Figure 2c) and lipid droplets (Figure 2d) were observed. Flow cytometry verified hSCAPs positively expressing mesenchymal markers (CD29, CD90, and CD44) while negatively expressing hematopoietic markers (CD34 and CD45) (Figure 2e).

Cell Counting Kit-8 (CCK-8) assay showed a significant decline in hSCAPs’ viability after 3–5 days of exposure to 10 and 100 µg/mL LPS compared to the control (0 µg/mL LPS) (Figure 2f). Wound healing assay revealed no notable difference in migration rates between the control (growth medium, GM) and LPS group (GM with 1 µg/mL LPS) (Figure 2g). Quantitative Real-Time Polymerase Chain Reaction (qRT-PCR) on day 3 confirmed a significant increase in pro-inflammatory cytokines’ mRNA levels in the LPS group (GM with 1 µg/mL LPS) compared to the control (GM) (Figure 2h). Consequently, we selected the concentration of 1 µg/mL LPS for subsequent experiments.

### 2.3. Downregulation of FTO Expression and Odontoblastic Differentiation Ability in hSCAPs under LPS Stimulation

**Figure 3:**
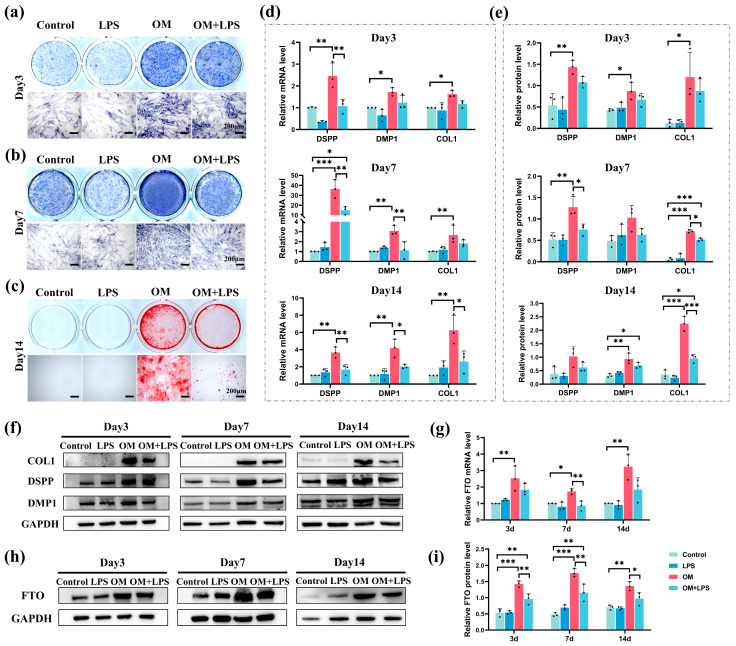
hSCAPs’ odontoblastic differentiation ability and FTO expression under different conditions. (**a**–**c**) ALP staining at 3 and 7 days showed decreased ALP activity, and ARS staining at day 14 indicated reduced mineralized nodules in the OM+LPS group compared to the OM group. (**d**) qRT-PCR revealed elevated mRNA levels of DSPP, DMP1, and COL1 in the OM group while showing decreased levels in the OM+LPS group. (**e**,**f**) Western blotting showed increased protein expression of DSPP, DMP1, and COL1 in the OM group, contrasting with decreased expression in the OM+LPS group. (**g**–**i**) qRT-PCR and Western blotting showed elevated FTO expression in OM, with reduced expression observed in OM+LPS. Control, growth medium (GM); LPS, GM with 1 µg/mL LPS; OM, odontoblastic induction medium; OM+LPS, OM with 1 µg/mL LPS; hSCAPs, human stem cells from the apical papilla; FTO, fat mass and obesity-associated protein; LPS, lipopolysaccharide; ALP, alkaline phosphatase; ARS, alizarin red S; qRT-PCR, Quantitative Real-Time Polymerase Chain Reaction; DSPP, dentin sialophosphoprotein; DMP1, dentin matrix protein-1; COL1, collagen I. * *p* < 0.05, ** *p* < 0.01, *** *p* < 0.001. Error bars: mean ± SD.

The hSCAPs were cultured in specific media: control (GM), LPS (GM with 1 µg/mL LPS), odontoblastic induction medium (OM), and OM+LPS (OM with 1 µg/mL LPS). Alkaline phosphatase (ALP) staining at 3 and 7 days showed that the OM and OM+LPS groups exhibited increased ALP activity compared to the control. However, LPS stimulation in the OM+LPS group reduced ALP activity compared to OM (Figure 3a,b). Alizarin red S (ARS) staining at day 14 showed no distinct mineralized nodules in the control and LPS groups, with a notable reduction in mineralized areas in the OM+LPS group relative to OM (Figure 3c). qRT-PCR and Western blotting showed no significant differences in the mRNA and protein levels of odontoblast marker genes (DSPP, DMP1, and COL1) between the control and LPS groups. In contrast, these genes were significantly higher in the OM group compared to the control. After 7 and 14 days of LPS stimulation, the mRNA and protein levels of DSPP, DMP1, and COL1 significantly decreased in the OM+LPS group compared to OM (Figure 3d–f). Furthermore, qRT-PCR and Western blotting confirmed the elevated mRNA and protein expression of FTO in the OM group compared to the control, while its expression significantly decreased in the OM+LPS group compared to OM (Figure 3g–i). These results indicate that LPS attenuates the odontoblastic differentiation ability and FTO expression of hSCAPs.

### 2.4. FTO Knockdown Impaired Odontoblastic Differentiation in hSCAPs

**Figure 4:**
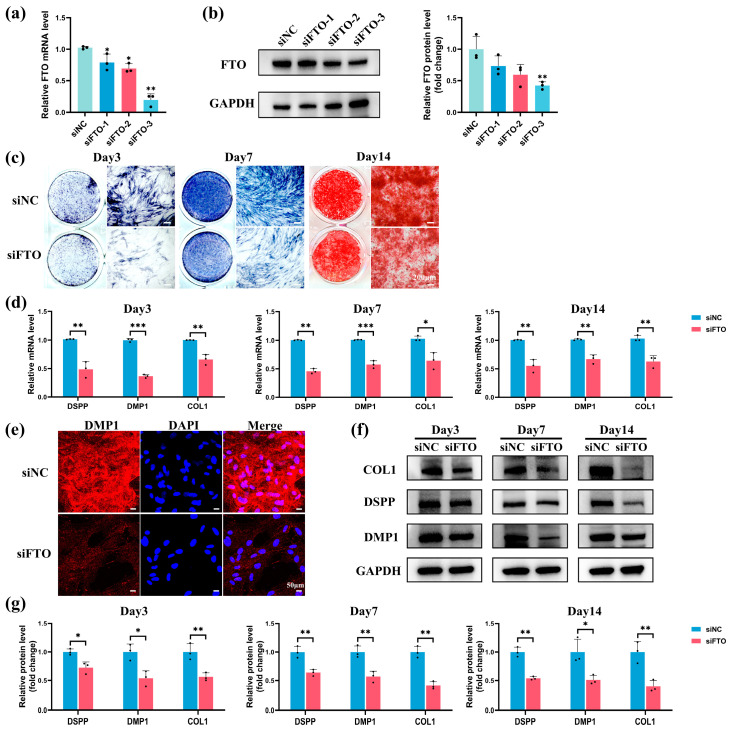
FTO knockdown impaired odontoblastic differentiation in hSCAPs. (**a**,**b**) qRT-PCR and Western blotting indicated that siFTO-3 exhibits the highest knockdown efficiency compared to siNC. (**c**) ALP staining at 3 and 7 days displayed reduced ALP activity, and ARS staining at day 14 revealed decreased mineralized nodules in the siFTO group. (**d**) qRT-PCR showed notably diminished DSPP, DMP1, and COL1 mRNA expression in the siFTO group. (**e**) IF showed a pronounced decline in anti-DMP1 staining in the siFTO group. (**f**,**g**) Western blotting confirmed significantly reduced DSPP, DMP1, and COL1 protein levels in the siFTO group relative to siNC. FTO, fat mass and obesity-associated protein; hSCAPs, human stem cells from the apical papilla; ALP, alkaline phosphatase; ARS, alizarin red S; qRT-PCR, Quantitative Real-Time Polymerase Chain Reaction; DSPP, dentin sialophosphoprotein; DMP1, dentin matrix protein-1; COL1, collagen I; IF, immunofluorescence. * *p* < 0.05, ** *p* < 0.01, *** *p* < 0.001. Error bars: mean ± SD.

FTO-targeting small interfering RNA (siRNA) (siFTO-1,2,3) samples were utilized to knock down FTO, with siNC as the negative control. siFTO-3 exhibited the highest knockdown efficiency at mRNA and protein levels 48 h and 72 h post-transfection, respectively (Figure 4a,b). Consequently, siFTO-3 was selected and designated as siFTO. The FTO-knockdown hSCAPs were subsequently transitioned to OM for further analysis. ALP staining at 3 and 7 days revealed a notable reduction in ALP activity in the siFTO group compared to siNC (Figure 4c). ARS staining at day 14 indicated diminished mineralized nodule formation in the siFTO group (Figure 4c). qRT-PCR showed significant downregulation of DSPP, DMP1, and COL1 mRNA expression in the siFTO group compared to siNC (Figure 4d). IF staining indicated a notable decrease in the anti-DMP1 staining levels in the siFTO group relative to siNC (Figure 4e). Western blotting consistently revealed significantly reduced DSPP, DMP1, and COL1 protein levels in the siFTO group compared to siNC (Figure 4f,g). These findings underscore that FTO knockdown compromises the odontoblastic differentiation ability of hSCAPs.

### 2.5. FTO Overexpression Rescued LPS-Induced Suppression of Odontoblastic Differentiation in hSCAPs

**Figure 5:**
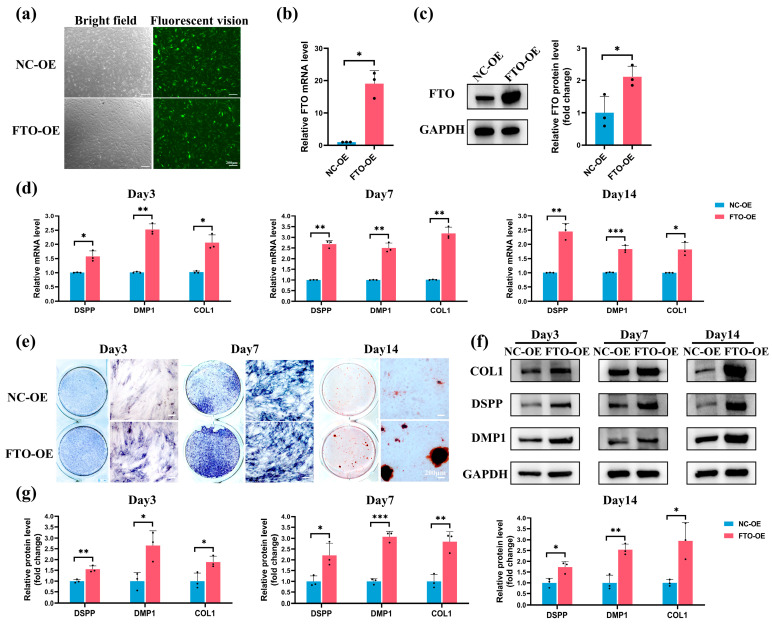
FTO overexpression rescued LPS-induced suppression of odontoblastic differentiation in hSCAPs. (**a**) Green fluorescent protein expression post-lentiviral transfection in hSCAPs. (**b**,**c**) qRT-PCR and Western blotting confirmed elevated FTO expression in the FTO-OE group. (**d**) qRT-PCR showed significantly increased DSPP, DMP1, and COL1 mRNA levels in FTO-OE. (**e**) ALP staining at 3 and 7 days displayed enhanced ALP activity, and ARS staining at day 14 indicated increased mineralized nodules in FTO-OE. (**f**,**g**) Western blotting validated notably elevated DSPP, DMP1, and COL1 protein levels in FTO-OE. FTO, fat mass and obesity-associated protein; LPS, lipopolysaccharide; hSCAPs, human stem cells from the apical papilla; qRT-PCR, Quantitative Real-Time Polymerase Chain Reaction; DSPP, dentin sialophosphoprotein; DMP1, dentin matrix protein-1; COL1, collagen I; ALP, alkaline phosphatase; ARS, alizarin red S. * *p* < 0.05, ** *p* < 0.01, *** *p* < 0.001. Error bars: mean ± SD.

The hSCAPs were transfected with lentivirus and divided into NC-OE (negative control) and FTO-OE (FTO overexpression) groups. Fluorescence imaging indicated that the transfected efficiency reached 80% (Figure 5a). qRT-PCR and Western blotting confirmed that FTO’s mRNA and protein levels significantly increased in the FTO-OE group compared to NC-OE (Figure 5b,c). FTO-overexpressing hSCAPs were then subjected to OM+LPS for subsequent experiments. qRT-PCR showed significantly increased DSPP, DMP1, and COL1 mRNA levels in the FTO-OE group compared to NC-OE (Figure 5d). ALP staining at 3 and 7 days revealed a pronounced increase in ALP activity in the FTO-OE group (Figure 5e). ARS staining indicated increased mineralized nodules in the FTO-OE group compared to NC-OE (Figure 5e). Consistently, Western blotting confirmed that the DSPP, DMP1, and COL1 protein levels were significantly elevated in the FTO-OE group compared to NC-OE (Figure 5f,g). The results indicated that FTO overexpression significantly rescued the LPS-induced downregulation of odontoblastic differentiation ability in hSCAPs.

### 2.6. FTO Modulated SMOC2 Expression during Odontoblastic Differentiation of hSCAPs

**Figure 6:**
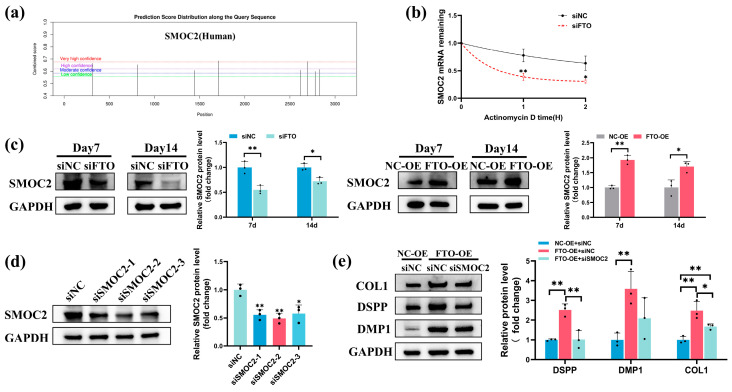
FTO promoted SMOC2 expression during odontoblastic differentiation of hSCAPs. (**a**) SRAMP identified eight potential m^6^A modification sites within the SMOC2 mRNA. (**b**) qRT-PCR showed significantly decreased SMOC2 mRNA stability in the siFTO group. (**c**) Western blotting illustrated that FTO knockdown decreased SMOC2 expression in hSCAPs cultured in OM; FTO overexpression increased SMOC2 protein levels in hSCAPs cultured in OM+LPS. (**d**) Western blotting confirmed that siSMOC2-2 exhibits the highest knockdown efficiency. (**e**) Western blotting revealed decreased DSPP and COL1 protein levels in the FTO-OE+siSMOC2 group. OM, odontoblastic induction medium; OM+LPS, OM with 1 µg/mL LPS; FTO, fat mass and obesity-associated protein; SMOC2, secreted modular calcium-binding protein 2; hSCAPs, human stem cells from the apical papilla; LPS, lipopolysaccharide; qRT-PCR, Quantitative Real-Time Polymerase Chain Reaction; DSPP, dentin sialophosphoprotein; DMP1, dentin matrix protein-1; COL1, collagen I. * *p* < 0.05, ** *p* < 0.01. Error bars: mean ± SD.

We further explored whether FTO modulates hSCAPs differentiation through SMOC2. SRAMP (http://www.cuilab.cn/sramp (accessed on 10 June 2023)) predicted eight potential m^6^A modification sites on the SMOC2 mRNA, with four showing heightened modification potential (Figure 6a). RPISeq (http://pridb.gdcb.iastate.edu/RPISeq/ (accessed on 27 June 2023)) indicated a significant interaction affinity between the FTO protein and SMOC2 mRNA (Table 1). qRT-PCR confirmed that SMOC2 mRNA stability markedly declined in the siFTO group relative to siNC (Figure 6b). Western blotting confirmed that FTO knockdown notably decreased SMOC2 protein expression in hSCAPs cultured in OM; FTO overexpression significantly elevated SMOC2 protein levels in hSCAPs cultured in OM+LPS (Figure 6c). Western blotting at 72 h post-transfection confirmed that siSMOC2-2 exhibits the highest knockdown efficiency, leading to its selection and designation as siSMOC2 (Figure 6d). Furthermore, Western blotting revealed a significant decrease in DSPP and COL1 protein expression in the FTO-OE+siSMOC2 group relative to FTO-OE+siNC when cultured in OM+LPS for 7 days (Figure 6e).

## 3. Discussion

To the best of our knowledge, this study represents the first investigation into the involvement of FTO in immature teeth with AP and its impact on the odontoblastic differentiation of hSCAPs. AP in young developing teeth is a critical global oral health concern among children. Distinct from adult teeth, the open apical foramen and fragile root canal wall complicate clinical intervention. Prior studies consistently report suboptimal outcomes, including recurrent periapical lesions, halted root formation, and intracanal obliteration [35,36,37]. Seeking and optimizing current strategies to preserve immature AP teeth remains a crucial clinical focus. Until now, the regulatory mechanisms of dentin formation, a central aspect of root development and formation, have not been fully elucidated. As the principal member of m^6^A demethylases, FTO profoundly affects cell homeostasis, metabolism, and stemness, which eventually regulate a wide array of bioprocesses and human diseases [38,39,40]. Recently, researchers have identified a functional link between dysregulation of m^6^A and tooth development. Methyltransferase-like 3 (METTL3) knockdown inhibits odontogenesis in dental roots by affecting nuclear factor I-C translation [41]. Conditional knockout of the demethylase alpha-ketoglutarate-dependent dioxygenase alkB homolog 5 (ALKBH5) in odontoblasts resulted in dysfunctional primary dentinogenesis [42]. Furthermore, FTO depletion resulting in dentine formation defects in mice has been reported [20]. Consistently, in an immature rat model with induced AP, our study identified significant downregulation of FTO around the infected root apex region, accompanied by diminished root length and expanded apical foramen. These findings suggest that reduced expression of FTO in the apical region may contribute to root development defects under inflammatory conditions.

Identifying the optimal stem cell source is critical in tissue engineering and regenerative medicine. In 2011, Lovelace et al. first elucidated that, during regenerative pulp procedures, bleeding allows mesenchymal stem cells (MSCs) to enter the root canal, identified as SCAPs due to their positive STRO-1 and CD105 markers [43]. Further research emphasizing the superior proliferative and mineralization potential, immunomodulatory effects, and early stem/progenitor cell characteristics of SCAPs underscores their indispensable role in advancing regenerative endodontics [44,45,46]. To achieve predictable dentin regeneration and repair, preserving or enhancing SCAPs’ vitality and pluripotency holds particular significance, especially considering their essential role in root development. Prior research has demonstrated that the prolonged inflammatory microenvironment inhibits osteogenic and dentinogenic differentiation of SCAPs [47]. Similarly, we observed a reduction in ALP activity, an early marker for odonto/osteogenic differentiation, on days 3 and 7 under LPS stimulation. Mineralized nodule formation, commonly used as an indicator of late-stage differentiation, decreased on day 14 under LPS stimulation. The DSPP gene is predominantly enriched and expressed in odontoblasts. DMP1, an acidic phosphorylated non-collagenous protein expressed in the dentin extracellular matrix, influences the odontogenic differentiation of precursor odontoblasts by controlling DSPP expression [48]. COL1 constitutes the principal organic matrix of dentin, synthesized and secreted by odontoblasts. In this study, we utilized these key markers to evaluate the odontoblastic differentiation ability of hSCAPs. We observed significant upregulation in DSPP, DMP1, and COL1 expression during odontoblastic induction while noting a declining trend after 7- and 14-day LPS stimulation. Additionally, FTO expression was considerably elevated during differentiation, contrasting with a marked decrease under LPS stimulation. The consistent alteration pattern observed in odontoblastic differentiation and FTO implies FTO’s potential positive regulatory function in hSCAPs differentiation.

Subsequently, we successfully constructed FTO-knockdown and FTO-overexpression hSCAPs, culturing them in an odontoblastic induction medium with or without LPS stimulation. During odontoblastic induction, FTO knockdown significantly compromised hSCAPs’ odontoblastic differentiation ability, as evidenced by attenuated ALP activity, reduced mineralized nodule formation, and downregulated expression of odontoblast markers (DSPP, DMP1, and COL1). Moreover, FTO overexpression notably enhanced the mineralization processes and marker expression levels under LPS-induced inflammation. Comprehensive gain- and loss-of-function studies confirmed that FTO enhances the odontoblastic differentiation ability of hSCAPs. Furthermore, our results indicate that LPS impedes the odontoblastic differentiation of hSCAPs through downregulating FTO, while FTO overexpression effectively mitigates the inhibitory effects induced by LPS. Several studies have reported the promotive role of FTO in cell differentiation, including bone marrow mesenchymal stem cells (BMSCs), cementoblasts, and DPSCs [20,21,22,23], aligning with our findings. Despite these observed roles, conflicting reports have also demonstrated the inhibitory effect of FTO on osteogenic differentiation [49,50]. Such discrepancies may stem from variances in the cellular milieu, complex regulatory cascades governing RNA-modifying enzymes, specific experimental contexts, and inherent biological heterogeneities. In this study, we revealed that FTO promotes the odontoblastic differentiation of hSCAPs in vitro under normal and inflammatory conditions.

SMOC2 regulates cell–cell and cell–microenvironment communication by interacting with matrix proteins, cell surface receptors, growth factors, and other bioactive effectors [51]. Prior extensive in vivo studies have substantiated the essential role of SMOC2 in root dentin formation [28,29,30,31,32]. Additionally, in vitro analyses have underscored the intricate interactions between SMOC2 and signaling molecules such as transforming growth factor-β1, Runx2, and bone morphogenetic proteins, pivotal for odonto/osteogenic differentiation [33,52]. Furthermore, pronounced expression of SMOC2 in the apical papilla growing region of developing mouse and human teeth, followed by a decrease in expression in adult teeth, has been reported [53,54]. In our study, bioinformatics analyses indicated a substantial interaction propensity between FTO and SMOC2, a finding validated by our results demonstrating that FTO knockdown during odontoblastic induction significantly reduced SMOC2 mRNA stability and protein expression. Under inflammatory conditions, FTO overexpression significantly elevated SMOC2 protein expression levels. Moreover, we found that knocking down SMOC2 in hSCAPs partially attenuated the promotion of odontoblastic differentiation ability mediated by FTO overexpression under LPS stimulation. These findings suggest a promotive role for the FTO/SMOC2 axis in regulating the odontoblastic differentiation of hSCAPs. Nonetheless, a more comprehensive investigation is imperative to decipher the intricate roles of SMOC2 in this regulatory network.

## 4. Materials and Methods

### 4.1. Animals

Sixteen male Sprague-Dawley juvenile rats (5 weeks old, weighing 100–120 g) were obtained from the Laboratory Animal Center of Sun Yat-sen University, China. Rats were anesthetized with an intraperitoneal injection of 2% pentobarbital sodium. Subsequently, a 1 mm diameter round bur was used to drill the mesial occlusal surface of the left mandibular first molars, exposing the pulp chamber, which was then directly exposed to the oral cavity. The untreated right mandibular first molars served as the control. Animals were provided with food and water in a 12 h light–dark cycle under specific pathogen-free (SPF) conditions. On days 7, 14, 21, and 28, four rats were randomly euthanized, and their mandibles were dissected and fixed with 4% paraformaldehyde (PFA, Beyotime, Shanghai, China) at 4 °C for 24 h.

### 4.2. Micro-Computed Tomography (micro-CT) Analysis

The mandibles were scanned utilizing a micro-CT scanner (μCT50, SCANCO, Brüttisellen, Switzerland) with the following parameters: 70 kV, 114 μA, and 8 μm resolution. Root lengths and the apical foramina diameter were measured using Mimics 21.0 software. The sagittal measurement plane was set to divide the mesial root equally through the apical foramen. For mesial root length measurement, a straight line was traced from the cement–enamel junction to the radiographic apex of the tooth. The diameter of the apical foramina was measured in the mesiodistal direction.

### 4.3. Histological Analysis

The samples underwent demineralization in 10% EDTA solution, followed by a gradient ethanol series dehydration. Subsequently, they were embedded in paraffin and sectioned at a thickness of 4 μm. After deparaffinization and rehydration, the sections were stained with hematoxylin–eosin (HE) and immunofluorescence (IF). The procedure followed the instructions (Servicebio, Wuhan, China) for HE staining, and images were captured using a slide scanner (Leica Aperio AT2, Wetzlar, Germany). The sections were subjected to antigen retrieval (Beyotime) and blocked with goat serum (Boster, Beijing, China) for IF staining. Subsequently, they were incubated with anti-FTO antibody (1:200, 27226-1-AP, Proteintech, Wuhan, China) overnight at 4 °C and incubated with fluorescence-labeled secondary antibody (1:300, E032420, EarthOx, Millbrae, CA, USA) for 1 h. Finally, the sections were mounted using the antifade mounting medium with DAPI (Beyotime) and observed under fluorescence microscope (Olympus FV3000, Tokyo, Japan).

### 4.4. Cell Culture and Identification

Human dental apical papilla tissue was obtained from healthy immature third molars extracted from individuals aged 16 to 18. After meticulous separation, the tissue was minced and digested with 3 mg/mL type I collagenase (Sigma-Aldrich, St. Louis, MO, USA) for 30 min at 37 °C. The tissue suspension was cultured in α-minimum essential medium (α-MEM, Gibco, Grand Island, NE, USA) supplemented with 20% fetal bovine serum (FBS, Bioind, Kibbutz Beit Haemek, Israel), 1% penicillin–streptomycin (Gibco), and 1% Gluta-Max (Gibco) to support cell growth. Then, cells were collected and maintained in a growth medium (GM) consisting of α-MEM with 10% FBS. Passages 3–4 were utilized for subsequent experiments.

Proliferation ability was evaluated via colony formation assay. hSCAPs were plated at 500 cells/well in 6-well plates and cultured in GM for 10 days. Subsequently, the cells were fixed using 4% PFA, stained with crystal violet (Beyotime), and observed under inverted fluorescence microscope (Zeiss Axio, Oberkochen, Germany).

To assess stemness surface markers, flow cytometry (Beckman Coulter, Brea, CA, USA) was employed with the following antibodies: anti-CD34, anti-CD45, anti-CD29, anti-CD44, and anti-CD90 (BioLegend, San Diego, CA, USA).

hSCAPs were cultured in either odontoblastic induction medium (OM), composed of GM, 10 mM β-glycerophosphate, 10 nM dexamethasone, and 50 μg/mL ascorbic acid (Sigma-Aldrich), or adipogenic induction medium, composed of GM, 200 μM indomethacin, 0.5 mM IBMX, 10 μg/mL insulin, and 1 μM dexamethasone (Sigma-Aldrich). The hSCAPs in the control group were cultured in GM. After 21 days of induction, the differentiation ability was assessed by staining for mineralized nodules using alizarin red and lipid droplets using oil red O (OriCell, Guangzhou, China). Images were captured using inverted fluorescence microscope (Zeiss).

### 4.5. Cell Viability and Migration

hSCAPs were seeded at a density of 2 × 10^3^ cells/well in 96-well plates and cultured overnight in GM. Escherichia coli LPS (O111:B4, Sigma-Aldrich) was dissolved in α-MEM (1 mg/mL) and then diluted in cell culture medium to the experimental concentration. Subsequently, the cells were exposed to LPS concentrations ranging from 0 to 100 µg/mL. Cell viability was assessed after 1, 3, and 5 days using the Cell Counting Kit-8 assay kit (CCK-8, Dojindo, Kumamoto, Japan). The optical density (OD) at 450 nm was measured using the microplate reader (Biotek Epoch2, Winooski, VT, USA).

For cell migration assay, hSCAPs were seeded in 6-well plates and cultured in GM until reaching 70–80% confluence. The wound scratch was created using a 200 µL sterile pipette tip. Subsequently, cells were incubated in GM containing 0 or 1 µg/mL LPS. Wound scratches were captured using inverted light microscope (Zeiss) after 24 h, and the scratch area was measured using Image J software (version 1.50). The wound healing rate was calculated as the difference between the initial (0 h) scratch area and the 24 h scratch area, divided by the initial (0 h) scratch area.

### 4.6. Alkaline Phosphatase (ALP) and Alizarin Red S (ARS) Staining

hSCAPs were seeded in 12-well plates and cultured in specific media: Control (GM), LPS (GM with 1 µg/mL LPS), OM, and OM+LPS (OM with 1 µg/mL LPS). ALP staining was performed on cells cultured for 3 and 7 days, fixed with 4% PFA, and stained with the BCIP/NBT ALP color development kit (Beyotime). ARS staining was performed on cells cultured for 14 days, fixed with 4% PFA, stained with alizarin red solution (OriCell), and captured using the inverted fluorescence microscope (Zeiss).

### 4.7. Cell Transfection and IF Staining

Lentivirus for human FTO overexpression and its corresponding control were designed and constructed by Hanbio (Shanghai, China). hSCAPs were transfected with lentivirus (MOI = 30) supplemented with 3 µg/mL polybrene (Hanbio). Then, stable transfected cells were selected using 1 μg/mL puromycin and passaged for further experiments. Small interfering RNA (siRNA) targeting *homo sapiens* FTO, SMOC2, and negative control were synthesized by GenePharma (Shanghai, China); their sequences were listed in Table S1. hSCAPs were transfected with 40 nM siRNAs, incorporating RNAFit (Hanbio), strictly following the manufacturer’s instructions.

For IF, transfected hSCAPs were cultured in OM for 3 days. Following this, cells were fixed with 4% PFA for 15 min, permeabilized with 0.5% Triton X-100 for 10 min (Beyotime), and blocked with goat serum for 30 min (Boster). They were then incubated overnight at 4 °C with anti-dentin matrix protein-1 antibody (DMP1, 1:200, sc-73633, Santa-Cruz Biotechnology, Santa Cruz, CA, USA), followed by a 50 min incubation with DyLight 594-conjugated secondary antibody (1:300, E032410-01, EarthOx). After mounting with the DAPI-containing antifade medium (Beyotime), cells were observed under fluorescence microscope (Olympus FV3000).

### 4.8. Quantitative Real-Time Polymerase Chain Reaction (qRT-PCR)

Following the instructions, the RNA was extracted using the RNA-easy Isolation Reagent (Vazyme, Nanjing, China). Subsequently, cDNA was synthesized using PrimeScript™ RT Master Mix (TaKaRa, Tokyo, Japan), and qRT-PCR was conducted with Hieff^®^ qPCR SYBR Green Master Mix (Yeasen, Shanghai, China) on the QuantStudio™ 7 Flex (Thermo Fisher Scientific, Waltham, MA, USA) following the instructions. Primer sequences for glyceraldehyde-3-phosphate dehydrogenase (GAPDH), tumor necrosis factor-alpha (TNF-α), interleukin-1beta (IL-1β), interleukin-6 (IL-6), dentin sialophosphoprotein (DSPP), DMP1, collagen I (COL1), FTO, and SMOC2 listed in Table S2. The expression levels of mRNA were calculated using the 2^−ΔΔCt^ method.

### 4.9. mRNA Stability Assay

Transfected hSCAPs were cultured in OM for 24 h. Cells were then exposed to 2 μg/mL actinomycin D (Boster) for 0, 1, and 2 h. Subsequently, total RNA was extracted, and qRT-PCR was conducted to quantify the relative level of SMOC2 mRNA.

### 4.10. Western Blotting

Cellular proteins were extracted using RIPA lysis buffer with 1% PMSF (Beyotime). The protein concentration was quantified using the bicinchoninic acid (BCA) protein assay kit (Cwbio, Beijing, China). Subsequently, 20 μg of proteins were separated by 4–12% SDS-PAGE (SurePAGE™, GenScript, Nanjing, China) and transferred onto 0.45 μm polyvinylidene fluoride (PVDF) membranes (Merck Millipore, Burlington, MA, USA). After blocking, the membranes were incubated overnight at 4 °C with primary antibodies, including anti-FTO (1:1000, 27226-1-AP, Proteintech), anti-DSPP (1:500, sc-73632, Santa-Cruz Biotechnology), anti-DMP1 (1:1000, DF8825, Affinity Biosciences, Changzhou, China), anti-COL1 (1:2000, ab6308, Abcam, Cambridge, UK), anti-SMOC2 (1:500, sc-376104, Santa-Cruz Biotechnology), and anti-GAPDH (1:1000; AF0006, Beyotime), followed by a 50 min incubation with corresponding secondary antibodies (1:2000, Beyotime). GAPDH was utilized as the internal control. Immunoblots were captured using the ChemiDoc Imaging System (Bio-Rad, Hercules, CA, USA), and densitometric analyses were performed using Image J software.

### 4.11. Statistical Analysis

The results, presented as the mean ± standard deviation (SD) of at least three individual experiments, underwent statistical analysis using SPSS 22.0 software. The unpaired Student’s *t*-test determined differences between the two groups, and multiple group comparisons were assessed through one-way ANOVA followed by Tukey’s post hoc test. *p* < 0.05 was considered statistically significant in all analyses.

## 5. Conclusions

Our findings revealed FTO involvement in the AP progression of young permanent teeth. LPS-induced inflammation impaired the odontoblastic differentiation ability of hSCAPs by downregulating FTO. FTO promoted hSCAPs’ odontoblastic differentiation under normal and inflammatory conditions, with the mechanism associated with alterations in SMOC2 expression. This study complements existing research on dentinogenesis and introduces a novel potential therapeutic target for AP in immature teeth.

## Figures and Tables

**Table 1:** RNA–protein interaction probabilities (RPISeq).

	SMOC2 Transcript Variant 1 mRNA	SMOC2 Transcript Variant 2 mRNA
Prediction using RF classifier	0.55	0.55
Prediction using SVM classifier	0.908	0.897

Table 1 Predictions with probabilities > 0.5 were considered positive.

## Data Availability

Data supporting reported results are available from the corresponding authors to all interested researchers.

## Supplementary Materials

The following supporting information can be downloaded at https://www.mdpi.com/article/10.3390/ijms25074045/s1.
