# Supplementary material for: FTO Positively Regulates Odontoblastic Differentiation via SMOC2 in Human Stem Cells from the Apical Papilla under Inflammatory Microenvironment
Source: Int J Mol Sci. 2024 Apr 5;25(7):4045. doi: 10.3390/ijms25074045 (PMC11012055; doi:10.3390/ijms25074045)
Supplement: Supplementary file 1 [file ijms-25-04045-s001.zip › ijms-2914301-supplementary.pdf]

FTO Positively Regulates Odontoblastic Differentiation via SMOC2 in Human Stem Cells from the Apical Papilla under Inflammatory Microenvironment

Qi Huang [1,2,3,†], Yumei Sun [1,2,3,†], Wushuang Huang [1,2,3], Fuping Zhang [1,2,3], Hongwen He [1,2,3], Yifan He [1,2,3,*] and Fang Huang [1,2,3,*]

1 Hospital of Stomatology, Sun Yat-sen University, Guangzhou 510055, China; huangq257@mail2.sysu.edu.cn (Q.H.); 13667453885@163.com (Y.S.); huangwsh26@mail.sysu.edu.cn (W.H.); zhangfp6@mail.sysu.edu.cn (F.Z.); hehw@mail.sysu.edu.cn (H.H.)

2 Guangdong Provincial Key Laboratory of Stomatology, Guangzhou 510055, China

3 Guanghua School of Stomatology, Sun Yat-sen University, Guangzhou 510055, China

* Correspondence: heyifan@mail2.sysu.edu.cn (Y.H.); hfang@mail.sysu.edu.cn (F.H.)

† These authors contributed equally to this work.

Supplementary Tables

Table S1. The target sequences for siRNA.

	**sequences (5' to 3')**
siFTO-1	GGACCUGGUUAGGAUCCAATT
siFTO-2	GCAGCAUACAACGUAACUUTT
siFTO-3	GGAUGACUCUCAUUCUCGAATT
siSMOC2-1	GCAAGUGUUCAUUCCUGAGTT
siSMOC2-2	GAAGAUAUUGCAUCACGUUTT
siSMOC2-3	GCAGCCCAAGAACGACAAUTT
siNC	UUCUCCGAACGUGUCACGUTT

Table S2. qRT-PCR primers.

**Gene**	**Forward primer**	**Reverse primer**
GAPDH	TCTCCTCTGACTTCAACAGCGACA	CCCTGTTGCTGTAGCCAAATTCGT
IL-1β	ATGATGGCTTATTACAGTGGCAA	GTCGGAGATTCGTAGCTGGA
IL-6	ACTCACCTCTTCAGAACGAATTG	CCATCTTTGGAAGGTTCAGGTTG
TNF-α	GAGGCCAAGCCCTGGTATG	CGGGCCGATTGATCTCAGC
FTO	TGATCTCAATGCCACCCACC	CAGAGCCAACTGACAGCGTT
DSPP	AAAGTGGTGTCCTGGTGCAT	CCTGGATGCCATTTGCTGTG
DMP1	CAGGAAGAGGTGGTGAGTGAGT	TGGATTCGCTGTCTGCTTGCT
COL1	AAAAGGAAGCTTGGTCCACT	GTGTGGAGAAAGGAGCAGAA
SMOC2	TTCTCGGCGCTCACGTTTTT	GTTGAAATTCACAACGGGAAAGG
